# Complex lipids shape cell growth and communication in moss

**DOI:** 10.1093/plphys/kiaf541

**Published:** 2025-10-25

**Authors:** Blanca Jazmín Reyes-Hernández

**Affiliations:** Assistant Features Editor, Plant Physiology, American Society of Plant Biologists; Faculty of Science, Department of Plant and Environmental Sciences, Section for Plant Glycobiology, University of Copenhagen, 1871 Frederiksberg C, Denmark

Biological membranes are mainly built from 3 classes of lipids: glycerophospholipids, sterols, and sphingolipids. Together, they provide structure, regulate fluidity, and create specialized domains for signaling and interactions. In plants, one of the most important group of sphingolipids is the glycosyl inositol phosphorylceramides (GIPCs). They are highly abundant in the plasma membrane and play unique structural and functional roles (Gronnier et al. 2016). Deficiency in GIPCs generally leads to visible growth defects, while plants completely missing GIPCs cannot be recovered (Rennie et al. 2014), showing that GIPCs are important for plant physiology and development.

GIPCs are a special type of plant lipid made of 3 main parts: a fatty tail, a small ring-shaped molecule called inositol, and one or more sugars attached (Fig.). The fatty part anchors the molecule into the membrane, while the inositol and sugars extend outward, where they can interact with the environment. Plants can decorate GIPCs with different sugars, sometimes 1 or 2, sometimes many, creating a remarkable variety of structures. This sugar diversity may allow GIPCs to perform specialized roles in different tissues. Yet, understanding their precise functions remains difficult because their biosynthesis is tightly linked to other sphingolipids, making it hard to separate the effects of GIPCs depletion from broader imbalances in lipid metabolism.

**Figure.**
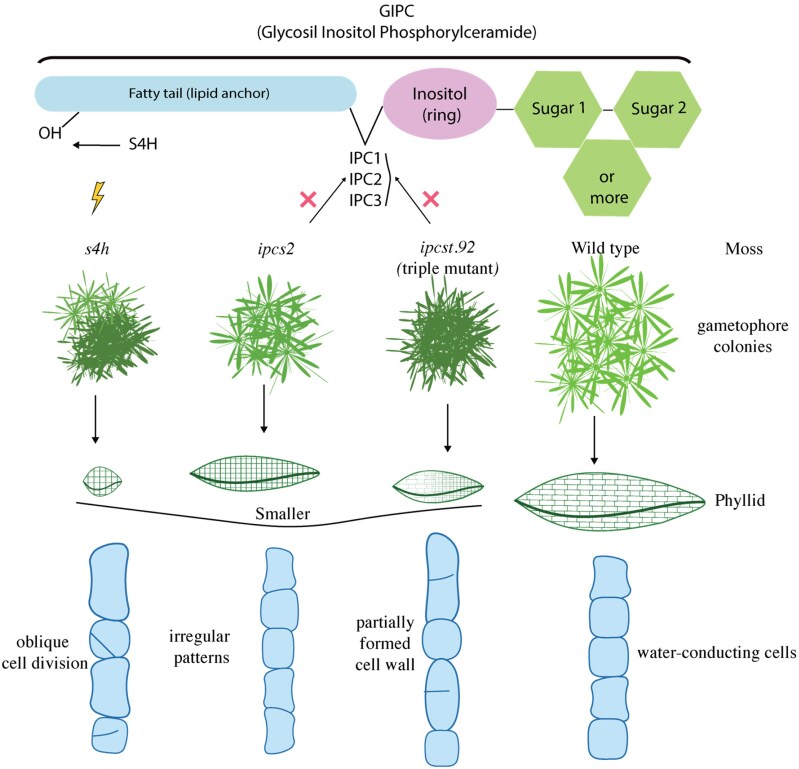
GIPCs regulate moss growth and cellular organization (based on Wegner et al. 2025 research). Membrane GIPCs are essential for multiple developmental traits in moss, including the overall size of gametophore colonies, the number of cells per phyllid, and the orientation of cell divisions. Mutants in IPCS (inositol phosphorylceramide synthase) and S4H (sphingoid base hydroxylase) show strong alterations in these processes, highlighting the importance of sphingolipid biosynthesis for proper colony architecture and cellular patterning. Together, these findings suggest that balanced GIPC production is a key determinant of moss morphology and growth.

To address this challenge, Wagner and collaborators (2025) recently reported in *Plant Physiology* the use of the moss *Physcomitrium patens* (*P. patens*) as a model to study GIPCs. The moss offers unique advantages such as making precise genetic edits without passing through a reproductive cycle, which allows the maintenance of mutants with severe developmental defects (Schaefer and Zrÿd 2001). The moss has a simple body, with filamentous protonema and single-cell-thick leaf-like phyllids, making it ideal for tracking cell division, differentiation, and growth. Lipid profiling has revealed a rich diversity of GIPCs in *P. patens*, including variants with different sugar types such as hexoses (Hex), N-acetyl hexoses (HexNAc), and pentoses (Pent) (Resemann et al. 2019). Together, these features make *P. patens* a powerful system for investigating the physiological functions of GIPCs.

Last year, Haslam et al. (2024) described several *P. patens* mutants affecting GIPCs biosynthesis. One was an *ipcs2* single mutant with reduced Hex-GIPCs, and another was a more severe triple mutant (*ipcst.92*, affected in *IPCS1*, *IPCS2*, and *IPCS3* genes) showing stronger losses of Hex-GIPCs than *ipcs2* and Hex-Hex-GIPCs. These mutants are affected in the enzyme Inositol Phosphorylceramide Synthase (IPCS), which produces the direct precursor of GIPCs, Inositol Phosphorylceramide (IPC). Interestingly, they also reported a third mutant named *s4h* (*sphinganine C4-hydroxylase*), altered in an enzyme that modifies the sphingoid base backbone (Fig.). This mutant accumulated 5 to 6 times more complex GIPCs (Gömann et al. 2021).

In their current work, the authors examined the growth of gametophore colonies (the leafy shoots) of those *P. patens* mutants and studied cell division in their leaf-like phyllids on their stem-like structure. They found strong defects in cell division in the triple *ipcst.92* and *s4h* mutants, as well as malformed plasmodesmata, the microscopic channels that connect the cytoplasm of neighboring cells (Zanini and Burch-Smith 2024). Because plasmodesmata control communication between cells, defects in their structure or maturation often disrupt developmental coordination and stress responses. These defects were further explored with assays of protein and molecule movement between cells, showing how sphingolipids are closely linked to fundamental aspects of cell biology.

In the wild-type moss, plasmodesmata normally mature from pore to branched form, and the 3 mutants analyzed by the authors failed to mature. Functional assays confirmed that these structural changes impacted transport. For example, in the triple mutant *ipcst.92* there was uncontrolled diffusion of molecules through incomplete walls, while *s4h* mutant restricted protein movement despite retaining plasmodesmata pore form. Together, these results highlight how GIPCs are critical for plasmodesmata maturation and cell-to-cell communication.

Lipid profiling performed on *ipcst2* and *ipcst.92* confirmed that as more disrupted IPCS isoforms are present in *P. patens*, the level of GIPCs decreases progressively; this correlates with the severity of phenotype. The same analysis of the *s4h* mutant showed that S4H enzyme is important to keep the balance of complex GIPCs (determined by number of sugars attached), which also leads to severe developmental abnormalities. These findings demonstrate that both the quantity and balance of GIPCs are essential for plant development.

Morphological analysis supported these findings. Using side-by-side growth assays of colonies, the authors showed that the *ipcs2* mutant had smaller colonies than the wild type, and the reduction in colony size was even more reduced in *ipcst.92* and *s4h* mutants (by 90%). Although all these mutants had dwarf, dense, and rounder shapes than wild type, interestingly, they presented more dry mass. Together, these findings reflected that altered sphingolipid metabolism does not simply regulate plant size but also determines colony shape, compactness, and structural integrity.

Further examination of phyllids revealed that the 3 mutants analyzed had fewer cells than wild type, while *ipcst.92* and *s4h* showed more severe defects. These mutants had less and shorter margin cells and had poorly developed conducting tissue, showing that GIPCs are needed for coordinated differentiation and organ formation. Transmission electron microscopy further highlighted defects like the incomplete walls with unanchored cell plates in the triple mutant *ipcst.92* and irregular callose-like deposits in *s4h.* Moreover, all mutants displayed plasma membrane detachment from the cell wall, with distinct patterns depending on the mutation.

In summary, this study shows that GIPCs are not only building blocks of membranes but also key players in regulating cell division, wall formation, and intercellular communication. By using *P. patens* as a complementary model to vascular plants, the work uncovers roles for sphingolipids that extend beyond what is known from studies of Arabidopsis. A particularly intriguing observation is that GIPCs-deficient moss mutants display defects like Arabidopsis mutants lacking PI4P (Lin et al. 2019), even though these lipids have been proposed to act in opposition (Ito et al. 2021). This proposed opposition raises important questions about how the balance between GIPCs and PI4P is maintained and how shifts in this equilibrium might influence other membrane lipids. Could differences in lineage-specific cell division mechanisms explain why moss and Arabidopsis respond differently to sphingolipid disruption? And do environmental cues reshape GIPC–PI4P relationships to adjust plant growth and signaling? Or even how exactly do GIPCs stabilize plasmodesmata during maturation? Addressing these questions will be essential to integrate lipid biochemistry with developmental outcomes and to understand how plants fine-tune connectivity across evolutionary scales.

## Data Availability

No additional data was used for the research described in the article.
